# Readability of AI-Generated Patient Information Leaflets on Alzheimer’s, Vascular Dementia, and Delirium

**DOI:** 10.7759/cureus.85463

**Published:** 2025-06-06

**Authors:** Jamila Tukur Jido, Ahmed Al-Wizni, Su Le Aung

**Affiliations:** 1 Department of Medicine for the Elderly, Queen Elizabeth University Hospital, Glasgow, GBR; 2 Department of Internal Medicine, Broomfield Hospital, Essex, GBR; 3 Department of Geriatric Medicine, Southend University Hospital NHS Foundation Trust, Southend-on-Sea, GBR

**Keywords:** alzheimer's disease, dementia vascular, hypoactive delirium, psychiatry of old age, readability analysis

## Abstract

Background

Large language models such as ChatGPT, DeepSeek, and Gemini are increasingly used to generate patient-facing medical content. While their factual accuracy has been explored, the readability of these outputs remains less well understood. Readability is a crucial component of health communication, particularly for older adults and those with lower health literacy. This study aimed to evaluate and compare the readability of patient information leaflets generated by three large language models - ChatGPT, DeepSeek, and Gemini - on the topics of Alzheimer's disease, vascular dementia, and delirium, using five validated readability metrics.

Materials and methods

We conducted a cross-sectional comparative study of patient information leaflets generated by three large language models on the topics of Alzheimer’s disease, vascular dementia, and delirium. Each model was prompted using identical queries, and the resulting texts were evaluated using five established readability metrics: Flesch Reading Ease, Flesch-Kincaid Grade Level, Gunning Fog Index, Simple Measure of Gobbledygook (SMOG) Index, and Automated Readability Index. Readability scores were compared using Kruskal-Wallis tests to identify statistically significant differences between models.

Results

ChatGPT consistently produced the most readable content, with the highest Flesch Reading Ease scores and the lowest grade-level indices. DeepSeek generated text that was markedly more complex and less accessible. Gemini performed intermediately, sometimes matching ChatGPT in specific indices but not consistently across all metrics. The difference in Flesch Reading Ease scores between models was statistically significant (H = 7.20, p = 0.027). Other metrics showed trends that approached significance.

Conclusions

There are meaningful differences in the readability of patient information generated by different large language models. ChatGPT appears to produce content that is more suitable for patient understanding, particularly in the context of older adult care. These findings highlight the need for careful evaluation of readability when using generative AI in clinical communication. Future research should incorporate expert review of content accuracy and appropriateness alongside readability.

## Introduction

Large language models (LLMs) are a class of artificial intelligence (AI) trained on diverse textual data, including books, articles, and websites, enabling them to understand and generate coherent human language [1]. These models can perform various language-related tasks, such as answering questions, summarising information, and translating text [2]. The use of LLMs has expanded into medical education, research, and patient communication [3]. Models such as ChatGPT, DeepSeek, and Gemini are now capable of producing detailed responses to medical enquiries, positioning them as potential adjuncts to traditional healthcare communication methods. ChatGPT is developed by OpenAI (San Francisco, USA) and trained on a wide range of internet text. DeepSeek (Hangzhou DeepSeek Artificial Intelligence Basic Technology Research Co., Ltd., Hangzhou, China) is a bilingual model designed for high-performance language tasks. Gemini, from Google (Mountain View, USA), integrates text and image processing for more comprehensive outputs [2,4].

Although research has primarily examined factual accuracy in AI-generated medical content [5-7], growing attention is now being paid to its linguistic accessibility. Without formal assessment, AI-generated material may inadvertently use complex or inaccessible language, undermining efforts to promote health literacy and patient-centred care [8]. Poorly readable materials are linked to reduced patient comprehension, lower health literacy, adverse outcomes, and increased healthcare costs, particularly among older adults and those with limited educational backgrounds [9-10]. Therefore, ensuring the accessibility of health information supports patient-centred care.

Major health organisations recommend presenting written patient information at or below a sixth-grade reading level. The American Medical Association (AMA) and United States National Institutes of Health (NIH) advise this standard to facilitate public understanding [11], while NHS England encourages plain language aimed at 11- to 12-year-olds [12]. Standard readability metrics, including the Flesch Reading Ease (FRE), Flesch-Kincaid Grade Level (FKGL), Gunning Fog Index, Simple Measure of Gobbledygook (SMOG) Index, and Automated Readability Index (ARI), are widely used to assess patient information leaflets and clinical guidelines [13,14]. However, their application to AI-generated content, particularly in geriatric psychiatry, remains unexplored.

This study evaluated the readability of outputs from ChatGPT, DeepSeek, and Gemini on three clinically significant topics in older-age psychiatry: Alzheimer’s disease, vascular dementia, and delirium. These topics were selected for their high prevalence in older adults, overlapping clinical features, and the well-established interrelationship between delirium and dementia. Delirium may accelerate cognitive decline in patients with underlying dementia, while dementia significantly increases the risk of developing delirium, underscoring the clinical importance of distinguishing and managing these conditions together [15]. By quantifying readability across models, this study aims to inform the development and application of LLMs in healthcare communication, ensuring they meet the needs of diverse patient populations. To our knowledge, this represents the first direct comparison of multiple LLMs within geriatric psychiatry.

## Materials and methods

This was a cross-sectional comparative study designed to assess and quantify the readability of outputs generated by three LLMs: ChatGPT (GPT-4), DeepSeek (V3), and Gemini (Flash 2.5). Each model was prompted with the standardised query: "Can you write me a patient information leaflet about [insert condition]?" for three clinical conditions: Alzheimer's disease, vascular dementia, and delirium. A purposive sampling approach was employed, selecting these conditions due to their high prevalence in older adults and overlapping clinical features, which make clear, accessible information particularly important. Identical prompts were used across all models to ensure consistency and minimise bias introduced by variations in query structure. Each model generated three outputs, resulting in a total of nine responses per model for subsequent analysis.

Each model was accessed via its official user interface between March and April 2025. The generated responses were copied without modification into a plain text format for analysis. Prior to readability analysis, all outputs were standardised to remove formatting elements that could influence text-based metrics. Specifically, hyperlinks, headings, and any bold or italicised text formatting were removed. Bullet points and numbered lists were converted into complete sentences where appropriate to maintain grammatical consistency. Special characters, such as asterisks and emojis (if present), were also removed. These steps were performed manually to ensure consistency across outputs and to enable accurate readability scoring based purely on text content. No formal sample size calculation was performed, given the exploratory nature of the study and the limited available outputs per model.

Readability was assessed using five validated metrics: Flesch Reading Ease (FRE), Flesch-Kincaid Grade Level (FKGL), Gunning Fog Index, Simple Measure of Gobbledygook (SMOG) Index, and Automated Readability Index (ARI). These tools are widely used in health communication research and offer complementary insights into sentence structure, word complexity, and the estimated reading level required for comprehension. The Flesch Reading Ease (FRE) score ranges from 0 to 100, with higher scores indicating more accessible text. The Flesch-Kincaid Grade Level (FKGL) estimates the U.S. school grade level required to understand the text. The Gunning Fog Index provides a similar estimate, with greater emphasis on complex and polysyllabic words. The SMOG Index, developed specifically for health-related materials, calculates grade level based on the number of polysyllabic words in a sample of sentences and is considered particularly reliable for assessing shorter medical texts. The Automated Readability Index (ARI) also produces a grade-level estimate, based on characters per word and words per sentence. Readability scores were calculated using Python (version 3.11) and the textstat package (version 0.7.3), which applies standardised formulas for each index.

Data availability

The datasets generated and analysed during the current study consist of non-identifiable text outputs from publicly available large language models (ChatGPT, DeepSeek, and Gemini). All responses were generated in April 2025 using the same prompt structure. The full outputs were collated and saved in a Word document format for subsequent readability analysis. These documents are available from the corresponding author on request.

Statistical analysis

Readability scores for each metric were treated as continuous variables. Non-parametric Kruskal-Wallis H tests were used to assess whether statistically significant differences in readability existed across the three large language models (ChatGPT, DeepSeek, and Gemini). This test was selected due to the small sample size (n = 3 per model per condition) and the non-normal distribution of the data. Each readability score corresponded to content generated by one model on a specific clinical topic (Alzheimer’s disease, vascular dementia, or delirium), yielding nine observations per metric. Post hoc pairwise comparisons were not performed, as the small sample size limited statistical power and the study was exploratory in nature. Statistical analysis was conducted using Python (version 3.11) with the textstat package (version 0.7.3), and a p-value < 0.05 was considered statistically significant. Results were visualised using grouped bar charts to support comparative interpretation across models and clinical topics.

Ethics statement

This study did not involve human participants, patient data, or animal subjects. All data analysed were generated by publicly accessible artificial intelligence tools. Ethical approval was therefore not required. No identifiable or sensitive information was included in the dataset.

## Results

Outputs generated by the three LLMs were assessed for readability across three clinical topics: Alzheimer's disease, vascular dementia, and delirium, using five standard indices. Comparative results are presented in tables. The combined readability scores are presented in the Appendices.

Alzheimer's disease

For content on Alzheimer’s disease (Table 1), ChatGPT produced text with the highest Flesch Reading Ease score (28.63), indicating comparatively better accessibility. Gemini followed with a score of 22.11, while DeepSeek’s score was lower at 11.57. DeepSeek also returned the highest grade levels across the Flesch-Kincaid (15.67), Gunning Fog (20.12), and ARI (14.25) indices. In contrast, Gemini had the lowest Flesch-Kincaid (13.46), Gunning Fog (18.05), SMOG (14.86), and ARI (11.74) scores, while ChatGPT produced intermediate values across most indices.

**Table 1:** Readability Scores for Alzheimer’s Disease Content Each value represents one output from a language model per topic (n = 3 per model). Data are presented as single numerical values derived from five readability metrics (FRE, FKGL, Gunning Fog Index, SMOG, ARI). FRE: Flesch Reading Ease; FKGL: Flesch-Kincaid Grade Level; SMOG: Simple Measure of Gobbledygook; ARI: Automated Readability Index

	Flesch Reading Ease	Flesch-Kincaid Grade Level	Gunning Fog Index	SMOG Index	Automated Readability Index (ARI)
ChatGPT (Alzheimer’s)	28.63	13.97	18.73	16.26	12.94
DeepSeek (Alzheimer’s)	11.57	15.67	20.12	16.56	14.25
Gemini (Alzheimer’s)	22.11	13.46	18.05	14.86	11.74

Vascular dementia

On the topic of vascular dementia (Table 2), ChatGPT again yielded the highest Flesch Reading Ease score (31.55), followed by Gemini (27.18). DeepSeek generated content with markedly lower readability (Flesch Reading Ease: 1.88) and the highest values across all other indices: Flesch-Kincaid Grade Level (22.09), Gunning Fog Index (27.33), SMOG Index (22.64), and ARI (23.26). This indicates that DeepSeek’s output was substantially more complex and likely requires an advanced reading level to comprehend. Gemini produced intermediate scores across most metrics, while ChatGPT achieved the lowest ARI (12.69), indicating the most accessible content overall for this topic.

**Table 2:** Readability Scores for Vascular Dementia Content Each value represents one output from a language model per topic (n = 3 per model). Data are presented as single numerical values derived from five readability metrics (FRE, FKGL, Gunning Fog Index, SMOG, ARI). FRE: Flesch Reading Ease; FKGL: Flesch-Kincaid Grade Level; SMOG: Simple Measure of Gobbledygook; ARI: Automated Readability Index

	Flesch Reading Ease	Flesch-Kincaid Grade Level	Gunning Fog Index	SMOG Index	Automated Readability Index (ARI)
ChatGPT (Vascular Dementia)	31.55	13.47	18.06	15.82	12.69
DeepSeek (Vascular Dementia)	-1.88	22.09	27.33	22.64	23.26
Gemini (Vascular Dementia)	27.18	14.68	19.61	16.98	13.68

Delirium 

For the delirium topic (Table 3), ChatGPT again demonstrated the highest readability with a FRE score of 33.01. It also returned relatively lower values for Gunning Fog (17.34), SMOG (15.45), and ARI (11.9). Gemini performed similarly, achieving the lowest ARI of any model across all conditions (10.51), and a FRE score of 26.68. DeepSeek once again produced the most complex content, with a FRE score of -6.19 and the highest grade-level and index values across the board: FKGL (20.74), Gunning Fog (25.99), SMOG (21.39), and ARI (21.53).

**Table 3:** Readability Scores for Delirium Content Each value represents one output from a language model per topic (n = 3 per model). Data are presented as single numerical values derived from five readability metrics (FRE, FKGL, Gunning Fog Index, SMOG, ARI). FRE: Flesch Reading Ease; FKGL: Flesch-Kincaid Grade Level; SMOG: Simple Measure of Gobbledygook; ARI: Automated Readability Index

	Flesch Reading Ease	Flesch-Kincaid Grade Level	Gunning Fog Index	SMOG Index	Automated Readability Index (ARI)
ChatGPT (Delirium)	33.01	13.51	17.34	15.45	11.9
DeepSeek (Delirium)	-6.19	20.74	25.99	21.39	21.53
Gemini (Delirium)	26.68	12.22	16.84	13.63	10.51

To assess whether there were statistically significant differences in readability scores between the three AI models, non-parametric Kruskal-Wallis tests were performed for each of the five readability indices. The raw data used for this is outlined in Tables 1-3. 

A statistically significant difference was found in Flesch Reading Ease (FRE) across the three models (H = 7.20, p = 0.027), indicating that at least one model generated text that was substantially more or less readable based on this measure. Although the other indices - Flesch-Kincaid Grade Level (FKGL), Gunning Fog Index, and Automated Readability Index (ARI) - did not reach statistical significance, they approached the conventional alpha level of 0.05 (p= 0.061, 0.066, and 0.061 respectively), suggesting possible trends that may be more evident with a larger sample. The SMOG Index showed the weakest evidence of a difference between models (H = 4.36, p = 0.113).

Overall, the findings suggest potential differences in readability between the outputs of ChatGPT, DeepSeek, and Gemini, with ChatGPT generally producing content that appears more accessible based on Flesch Reading Ease scores. However, due to the small sample size, lack of post hoc testing, and absence of effect size reporting, these observations should be interpreted as exploratory trends rather than definitive comparisons. Further research with larger samples, formal pairwise comparisons, and practical significance metrics will be necessary to draw more robust conclusions about model performance.

These findings are summarised in Table 4, which outlines the results of the Kruskal-Wallis tests for each readability measure.

**Table 4:** Kruskal-Wallis Test Results Comparing Readability Scores Across AI Models Kruskal-Wallis H test results for each readability metric comparing outputs from ChatGPT, DeepSeek, and Gemini. A p-value < 0.05 was considered statistically significant.

	Kruskal-Wallis H	p-value
Flesch Reading Ease	7.2	0.027
Flesch-Kincaid Grade Level	5.6	0.061
Gunning Fog Index	5.422	0.066
SMOG Index	4.356	0.113
Automated Readability Index (ARI)	5.6	0.061

Graphical interpretation of readability trends

The grouped bar charts provide a comparative visualisation of the five readability metrics across the three LLMs for each clinical topic. These figures support the numerical results presented in the tables and illustrate consistent patterns in readability performance.

**Figure 1:**
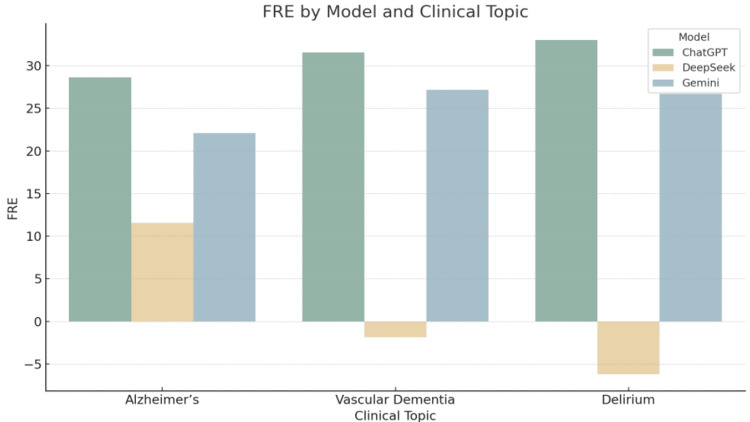
Flesch Reading Ease by Model and Clinical Topic Grouped bar chart comparing Flesch Reading Ease (FRE) scores for patient information leaflets generated by ChatGPT, DeepSeek, and Gemini across three clinical topics: Alzheimer’s disease, vascular dementia, and delirium. FRE scores range from 0 to 100, with higher values indicating greater readability.

Figure 1 demonstrates that ChatGPT consistently achieved the highest FRE scores, indicating easier-to-read text across all topics. In contrast, DeepSeek scored lowest, especially for vascular dementia and delirium, where FRE values were negative. These negative scores imply language that would likely be inaccessible to the average reader.

**Figure 2:**
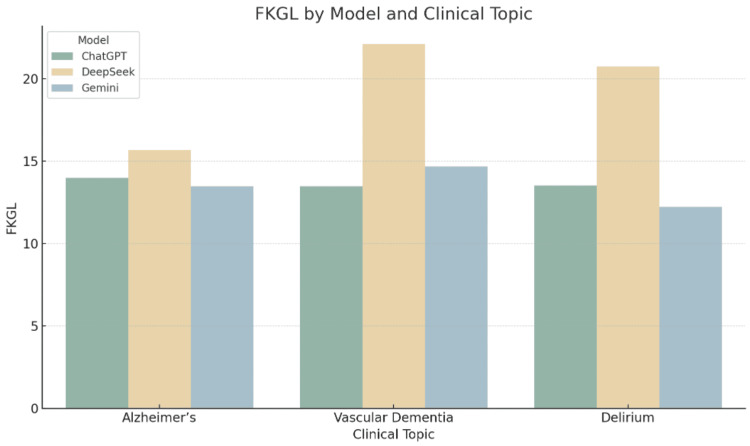
Flesch-Kincaid Grade Level by Model and Clinical Topic Grouped bar chart illustrating the Flesch-Kincaid Grade Level (FKGL) scores for patient information leaflets generated by ChatGPT, DeepSeek, and Gemini across three clinical topics: Alzheimer’s disease, vascular dementia, and delirium. FKGL estimates the U.S. school grade level required to comprehend the text, with higher values indicating increased complexity.

Figure 2 shows that DeepSeek generated content requiring the highest reading levels according to the Flesch-Kincaid Grade Level. This suggests that readers may need post-secondary or even university-level education to fully comprehend the text. ChatGPT and Gemini scored notably lower, falling closer to the sixth-form level.

**Figure 3:**
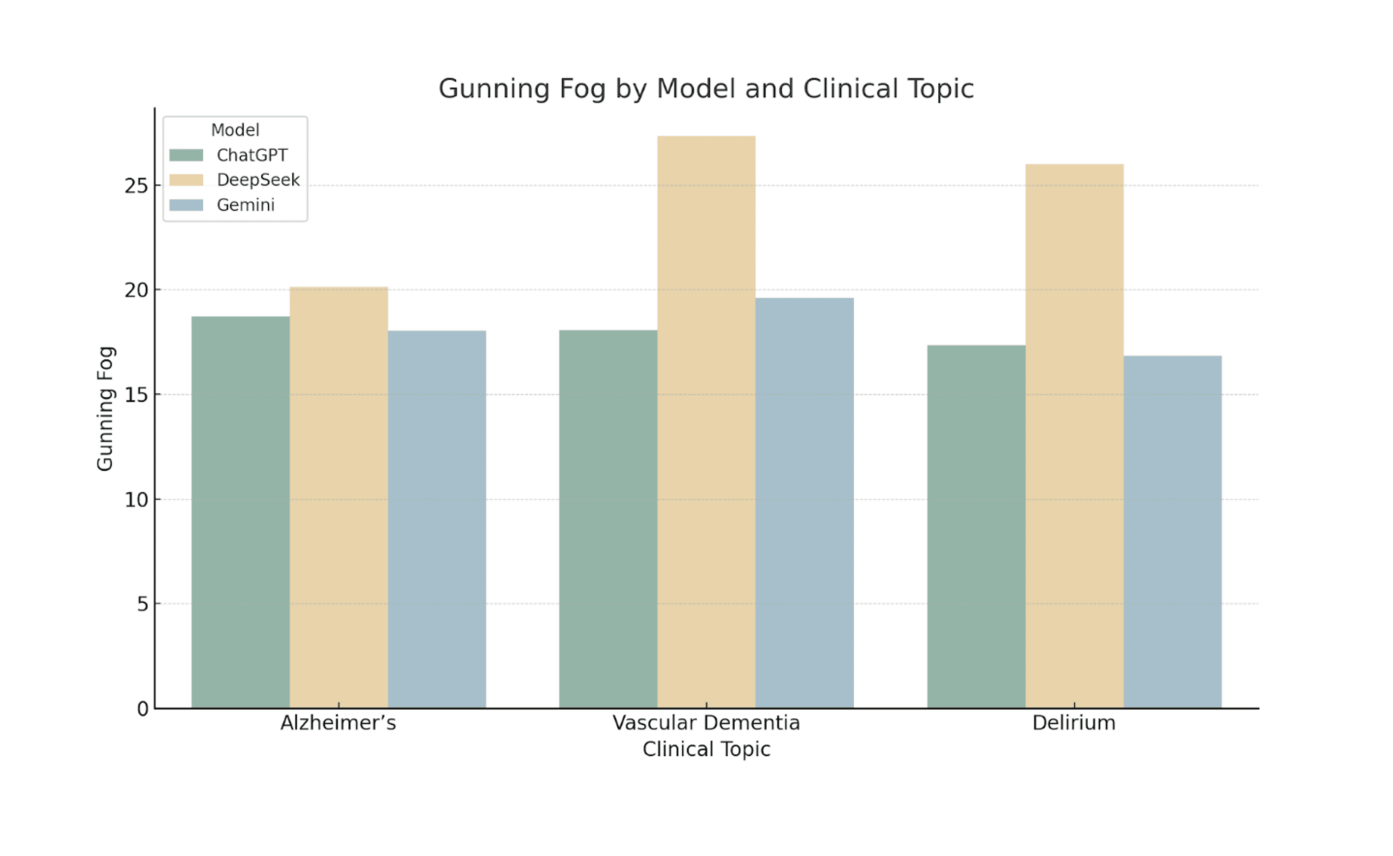
Gunning Fog by Model and Clinical Topic Grouped bar chart showing Gunning Fog Index scores for patient information leaflets generated by ChatGPT, DeepSeek, and Gemini across three clinical topics: Alzheimer’s disease, vascular dementia, and delirium. The Gunning Fog Index estimates the years of formal education required to understand a piece of text. Higher scores indicate greater linguistic complexity.

Figure 3 reveals a similar pattern using the Gunning Fog Index. DeepSeek’s outputs consistently required more years of formal education compared to the other two models. The difference was particularly striking for vascular dementia, where the Gunning Fog score exceeded 27.

**Figure 4:**
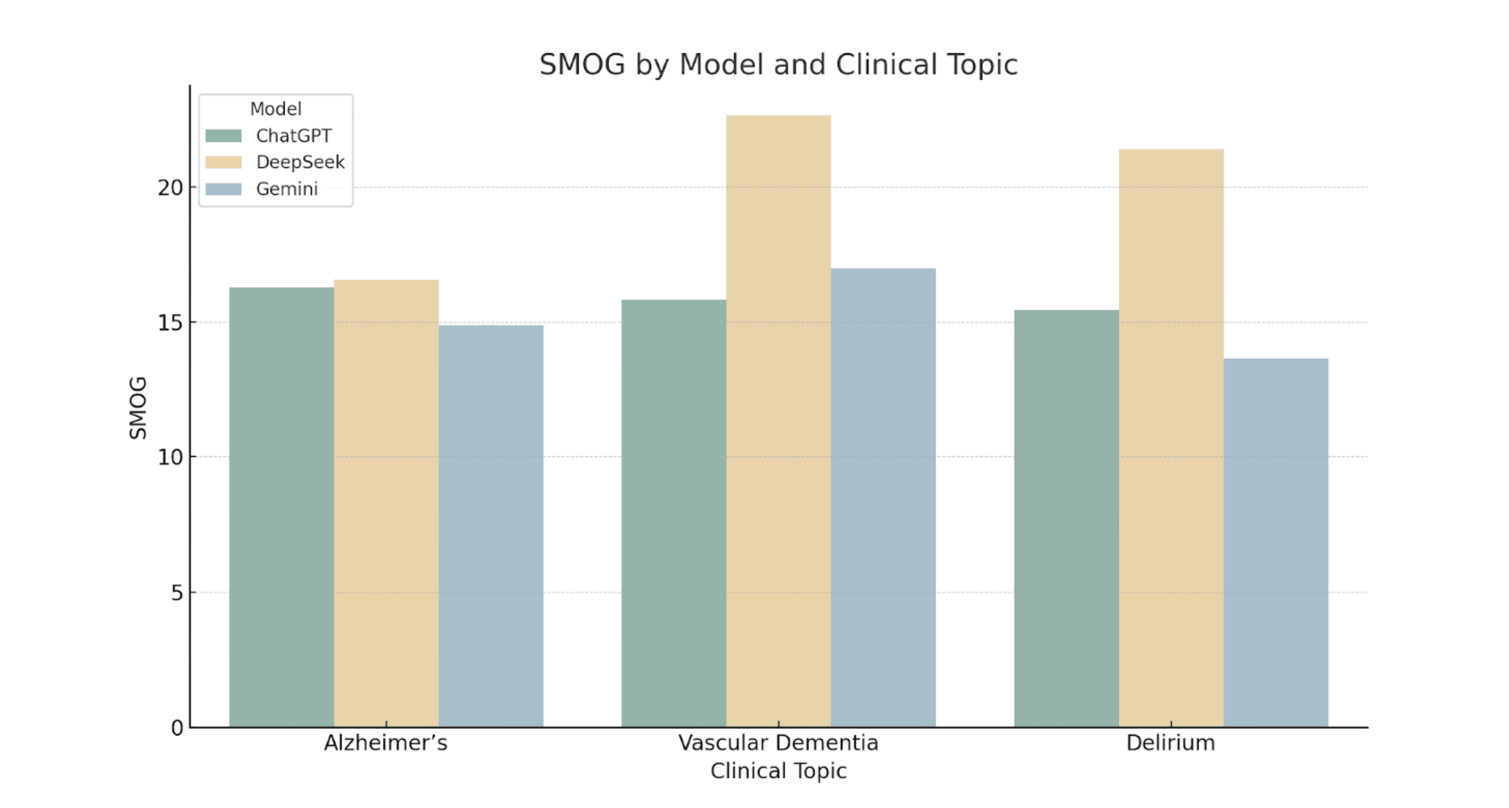
SMOG by Model and Clinical Topic Grouped bar chart displaying SMOG (Simple Measure of Gobbledygook) Index scores for patient information leaflets generated by ChatGPT, DeepSeek, and Gemini across three clinical topics: Alzheimer’s disease, vascular dementia, and delirium. The SMOG Index estimates the U.S. school grade level required to understand a text, based on the number of polysyllabic words. Higher values indicate increased complexity.

Figure 4 highlights the SMOG Index results. While differences were less pronounced, DeepSeek again produced the most linguistically complex outputs, particularly for delirium. Gemini had the lowest SMOG values in some cases, although ChatGPT’s scores remained close.

**Figure 5:**
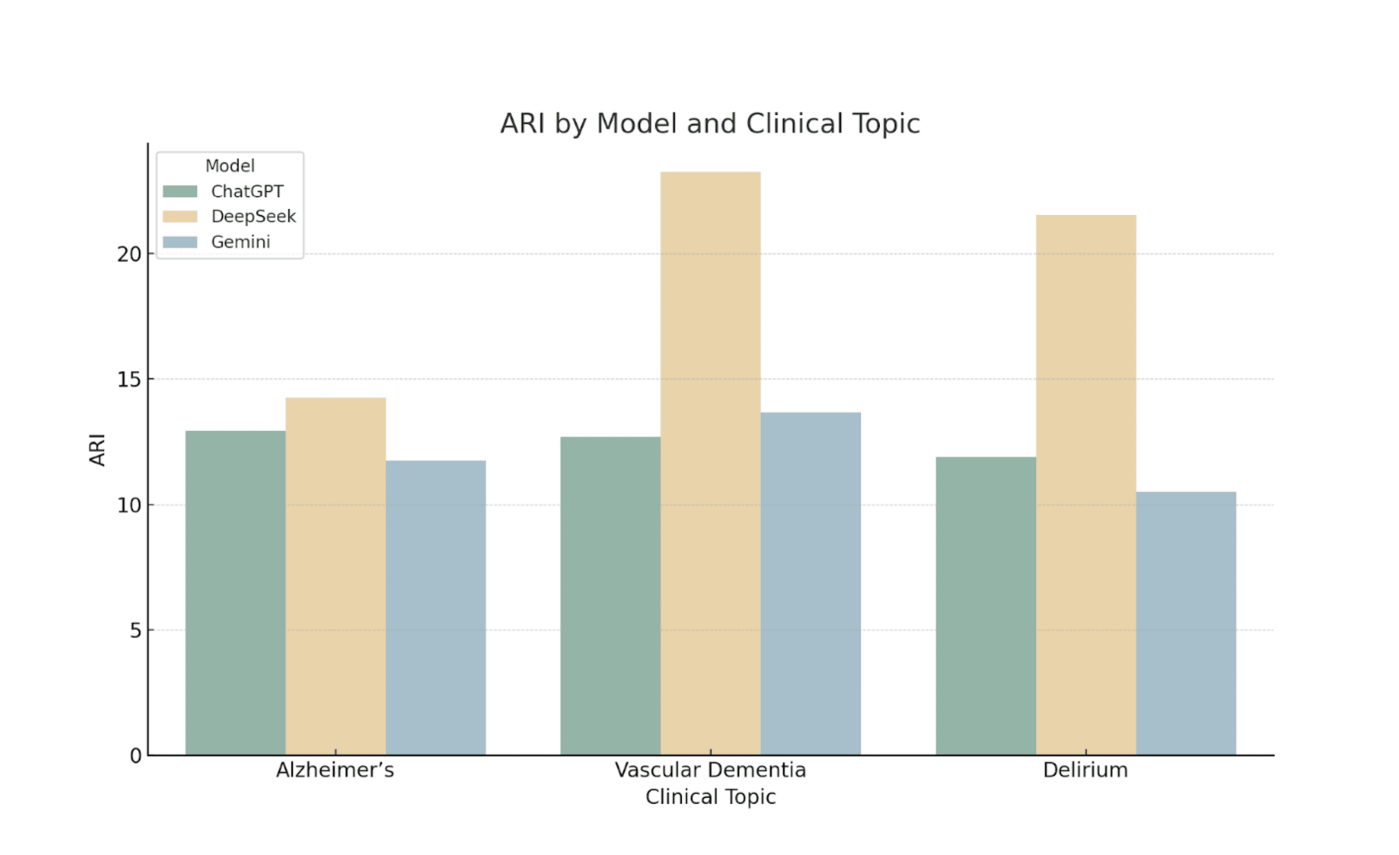
ARI by Model and Clinical Topic Grouped bar chart comparing Automated Readability Index (ARI) scores for patient information leaflets generated by ChatGPT, DeepSeek, and Gemini across the topics of Alzheimer’s disease, vascular dementia, and delirium. The ARI estimates the U.S. school grade level needed to understand the text, based on characters per word and words per sentence. Higher scores indicate more complex and less accessible language.

Figure 5 presents the ARI findings. DeepSeek returned ARI values above 20 for both vascular dementia and delirium, reinforcing the impression of reduced accessibility. In comparison, ChatGPT’s and Gemini’s values fell within a more readable range, often below 13.

Taken together, these bar charts reinforce the statistical findings, demonstrating that ChatGPT tends to produce more readable patient information leaflets across a range of clinically relevant topics. The visual separation between models - particularly in grade-level indices - emphasises the potential implications for patient comprehension and health literacy when different AI models are used to generate content.

Summary of trends

Across all three topics, ChatGPT consistently generated the most readable text as indicated by Flesch Reading Ease and comparatively lower readability grade indices. DeepSeek’s content was more complex, with higher grade-level scores and lower ease-of-reading metrics, particularly in the domains of vascular dementia and delirium. Gemini generally performed intermediately, sometimes achieving the lowest ARI values but not consistently outperforming ChatGPT across the board.

## Discussion

To our knowledge, this is the first study to evaluate the readability of LLM-generated responses on clinical topics relevant to old age psychiatry. Outputs were assessed using five established readability metrics to quantify linguistic complexity and accessibility. Marked variability between models was observed. ChatGPT consistently achieved the highest FRE scores across all three topics, indicating the most accessible responses. By contrast, DeepSeek generated negative FRE values for vascular dementia and delirium, suggesting the use of highly technical language. Gemini produced outputs that were more readable than DeepSeek’s but less so than ChatGPT’s, occupying an intermediate position.

Statistical analysis supported these findings. A Kruskal-Wallis test demonstrated a significant difference in FRE scores between models (H = 7.20, p = 0.027), reinforcing ChatGPT’s relative readability. Although differences in FKGL, Gunning Fog, and ARI did not reach statistical significance, they approached conventional thresholds (p = 0.061-0.066), suggesting a trend towards greater complexity in DeepSeek’s outputs. The SMOG Index showed minimal variation (p = 0.113), indicating comparable polysyllabic word use across models. Collectively, these results highlight meaningful differences in sentence structure and linguistic accessibility. While statistically significant and near-significant results were reported, the study did not calculate effect sizes. As such, the magnitude and clinical relevance of these differences remain uncertain and should be addressed in future studies.

These differences carry important clinical implications. Readability is one component of effective public health communication, as it influences initial comprehension and engagement [16]. While ChatGPT’s relatively higher readability scores suggest greater accessibility for general audiences, readability alone does not guarantee understanding, particularly for patients with cognitive impairment or low health literacy. Prior studies have shown that even linguistically simplified LLM-generated content may be pitched at overly high reading levels or lack sufficient clinical depth. For example, ChatGPT’s responses to lung cancer queries often aligned with university-level complexity (FRE 40.5), raising accessibility concerns [17]. Similarly, AI-generated materials in foot and ankle surgery and anaesthesia have been found lacking in completeness and nuance compared to official sources [18,19].

These findings reinforce the need for human oversight and caution against over-reliance on readability metrics alone. As readability does not assess contextual accuracy, emotional tone, or true patient comprehension, future studies should incorporate direct patient feedback, usability testing, and clinician review to ensure that AI-generated content is both understandable and appropriate.

Health literacy is a well-established determinant of clinical outcomes, with lower literacy linked to delayed diagnoses, poorer prognosis, and increased healthcare costs [20,21]. Inadequately tailored AI-generated information could exacerbate these inequalities. Future iterations of LLMs capable of dynamically adjusting linguistic complexity based on user demographics or preferences may help mitigate this risk. As such, accessibility should be considered central to responsible AI deployment in healthcare communication.

While readability is an important factor contributing to overall health literacy, it primarily reflects the structural and lexical complexity of text, rather than a patient's actual understanding or ability to apply health information. This study focuses specifically on the quantitative assessment of readability using validated indices. We acknowledge that readability alone does not equate to comprehension, particularly for individuals with cognitive impairment, and that further research is needed to evaluate the comprehensibility and clinical utility of AI-generated health content [22,23].

This study has limitations. Readability metrics offer only a quantitative proxy for text complexity and do not capture factors such as syntactic clarity, coherence, tone, or factual accuracy. While these indices are widely used in health communication research, they are unable to assess true comprehension or the contextual appropriateness of medical content. Furthermore, the analysis was confined to three geriatric conditions, limiting the generalisability of the findings across broader clinical areas. The use of a small sample size (n = 3 per model per condition) also restricts statistical power and precludes more granular comparisons through post-hoc testing or effect size analysis. Only a single prompt per model per topic was used, which does not reflect the potential variability in AI-generated outputs across repeated interactions. Additionally, the study did not include subjective human evaluation, which could have provided complementary insights into the perceived clarity, accuracy, and usefulness of the content. Future research should expand on these findings using qualitative methodologies, including patient and clinician surveys, focus groups, and cognitive interviews, to assess comprehension and utility in real-world contexts. The potential for adaptive large language models that dynamically adjust complexity based on user demographics or cognitive needs also warrants exploration. Finally, prospective studies are needed to evaluate the impact of AI-generated patient information on understanding, behaviour, adherence, and healthcare utilisation.

## Conclusions

This study identifies significant variation in the readability of AI-generated patient information produced by large language models. Descriptive and statistical findings suggest that ChatGPT tends to generate more accessible text based on Flesch Reading Ease, while DeepSeek outputs were consistently more complex. These patterns, though based on a small sample and without post hoc testing, may have implications for public health communication, where linguistic accessibility supports patient engagement and potential understanding.

However, readability formulas provide only a surface-level proxy for text complexity and do not account for comprehension, accuracy, or clinical appropriateness, especially critical for older adults and cognitively impaired populations. More readable text may inadvertently oversimplify nuanced medical information. Accordingly, future research must move beyond readability scores to evaluate how different patient groups interpret AI-generated materials, incorporating qualitative methods and user testing to assess real-world impact on understanding, adherence, and informed decision-making.

## Appendices

**Table 5:** Combined Readability Scores Table 5 presents the combined readability scores for patient information leaflets generated by each large language model (ChatGPT, DeepSeek, and Gemini) on the topics of Alzheimer’s disease, vascular dementia, and delirium. The readability metrics include Flesch Reading Ease (FRE), Flesch-Kincaid Grade Level (FKGL), Gunning Fog Index, Simple Measure of Gobbledygook (SMOG) Index, and Automated Readability Index (ARI). Each row corresponds to a specific model and topic pairing. These scores were treated as continuous variables and used to perform Kruskal–Wallis H tests to determine whether statistically significant differences in readability existed between the models. A p-value less than 0.05 was considered statistically significant.

Condition (Model)	Flesch Reading Ease	Flesch-Kincaid Grade Level	Gunning Fog Index	SMOG Index	Automated Readability Index
Alzheimer's (ChatGPT)	28.63	13.97	18.73	16.26	12.94
Alzheimer's (Deepseek)	11.57	15.67	20.12	16.56	14.25
Alzheimer's (Gemini)	22.11	13.46	18.05	14.86	11.74
Vascular Dementia (ChatGPT)	31.55	13.47	18.06	15.82	12.69
Vascular Dementia (Deepseek)	-1.88	22.09	27.33	22.64	23.26
Vascular Dementia (Gemini)	27.18	14.68	19.61	16.98	13.68
Delirium (ChatGPT)	33.01	13.51	17.34	15.45	11.9
Delirium (Deepseek)	-6.19	20.74	25.99	21.39	21.53
Delirium (Gemini)	26.68	12.22	16.84	13.63	10.51
